# Modeling the costs and long-term health benefits of screening the general population for risks of cardiovascular disease: a review of methods used in the literature

**DOI:** 10.1007/s10198-015-0753-2

**Published:** 2015-12-18

**Authors:** David Epstein, Leticia García-Mochón, Stephen Kaptoge, Simon G. Thompson

**Affiliations:** 1 Department of Applied Economics, University of Granada, Campus de la Cartuja, 18071 Granada, Spain; 2 Escuela Andaluza de Salud Pública, Granada, Spain; 3 Department of Public Health and Primary Care, University of Cambridge, Cambridge, UK

**Keywords:** Cost-effectiveness analysis, Screening, Cardiovascular disease, Primary prevention, Statins, Literature review, I180, H510

## Abstract

**Background:**

Strategies for screening and intervening to reduce the risk of cardiovascular disease (CVD) in primary care settings need to be assessed in terms of both their costs and long-term health effects. We undertook a literature review to investigate the methodologies used.

**Methods:**

In a framework of developing a new health-economic model for evaluating different screening strategies for primary prevention of CVD in Europe (EPIC-CVD project), we identified seven key modeling issues and reviewed papers published between 2000 and 2013 to assess how they were addressed.

**Results:**

We found 13 relevant health-economic modeling studies of screening to prevent CVD in primary care. The models varied in their degree of complexity, with between two and 33 health states. Programmes that screen the whole population by a fixed cut-off (e.g., predicted 10-year CVD risk >20 %) identify predominantly elderly people, who may not be those most likely to benefit from long-term treatment. Uncertainty and model validation were generally poorly addressed. Few studies considered the disutility of taking drugs in otherwise healthy individuals or the budget impact of the programme.

**Conclusions:**

Model validation, incorporation of parameter uncertainty, and sensitivity analyses for assumptions made are all important components of model building and reporting, and deserve more attention. Complex models may not necessarily give more accurate predictions. Availability of a large enough source dataset to reliably estimate all relevant input parameters is crucial for achieving credible results. Decision criteria should consider budget impact and the medicalization of the population as well as cost-effectiveness thresholds.

**Electronic supplementary material:**

The online version of this article (doi:10.1007/s10198-015-0753-2) contains supplementary material, which is available to authorized users.

## Introduction

Cardiovascular disease (CVD) is a major public health problem with a huge impact on health service budgets in European countries [1]. Current guidelines for primary prevention of CVD generally involve a combination of advice for lifestyle change and/or pharmacological intervention (e.g., statins or anti-hypertensives) in those assessed to be at sufficiently high-risk [2–5]. The parameters of such programmes vary greatly between countries. Most countries use opportunistic case finding, although the UK has recently launched a national screening programme [6]. National guidelines recommend initiating statin therapy when the 10-year risk of CVD exceeds 7.5 % in the USA [2], 10 % in the UK [7], and 20 % in other countries [8]. An explicit comparison of the costs and benefits of CVD risk assessment and treatment informs some guidelines [7], but not others [5]. Cost-effectiveness of a screening strategy might be optimized by appropriate choice of the risk algorithm, employing the most efficient threshold for initiating treatment [9], or using stepwise or targeted screening strategies [10]. There are also concerns about the long-term side effects of statins and medicalizing a large proportion of the general population [11].

In this paper, we report a literature review conducted to help develop a new health-economic model for evaluating different screening strategies and interventions to prevent CVD in European countries (http://www.epiccvd.eu). We identify a series of questions that an economic analysis in this area ought to address, and describe and comment on the approaches used. These questions are based on the authors’ experience and discussions while preparing the paper. Several published reviews of the health-economic evidence for primary prevention of CVD already exist [12–17]. Each offers useful insights, but none considers all of the following methodological questions that we believe need to be addressed together:

1. What are the criteria used for cost-effectiveness?
2. What is the structure of the economic model?
3. What are the population and strategies of interest?
4. How are primary CVD outcomes defined and assessed?
5. How are individuals at high risk of CVD identified and treated?
6. How are resources, costs and quality of life measured?
7. How is the model implemented and validated?

The structure of the paper is as follows. First, we describe the literature search. Second, we discuss the health-economic approaches used to address each question in the selected articles. We compare and critique these approaches as we go. Lastly, we discuss some general themes raised by the review and tentatively propose some recommendations. The recommendations reflect our opinion, but are intended to summarize the advantages and drawbacks of each approach in different decision contexts.

## Literature search

We conducted a literature review to identify studies describing health-economic models of cost-effectiveness of screening strategies for primary prevention of CVD in the general population. The web appendix (eTable 1) provides details of the bibliographic terms used and the search results obtained from PubMed and Web of Science databases. Studies were included in the final review if they were published between January 2000 and September 2013, concerned CVD screening strategies or general health checks that could be implemented in a primary care setting with current technology, were full economic evaluations (i.e., include both costs and benefits), targeted the adult general population without previous history of CVD, and were based on models with a time horizon >1 year. Studies were excluded if they assessed tests or technology not commonly available in primary care settings in western Europe, did not include CVD screening as the initial step (e.g., economic evaluation of statin treatments), or were targeted at sub-groups of the general population (e.g., people already identified as intermediate risk, or patients with diabetes mellitus). As this paper is a review of methodological approaches, rather than a quality assessment of the articles themselves, we also excluded articles that replicated broadly similar methods to another included study.

**Fig. 1:**
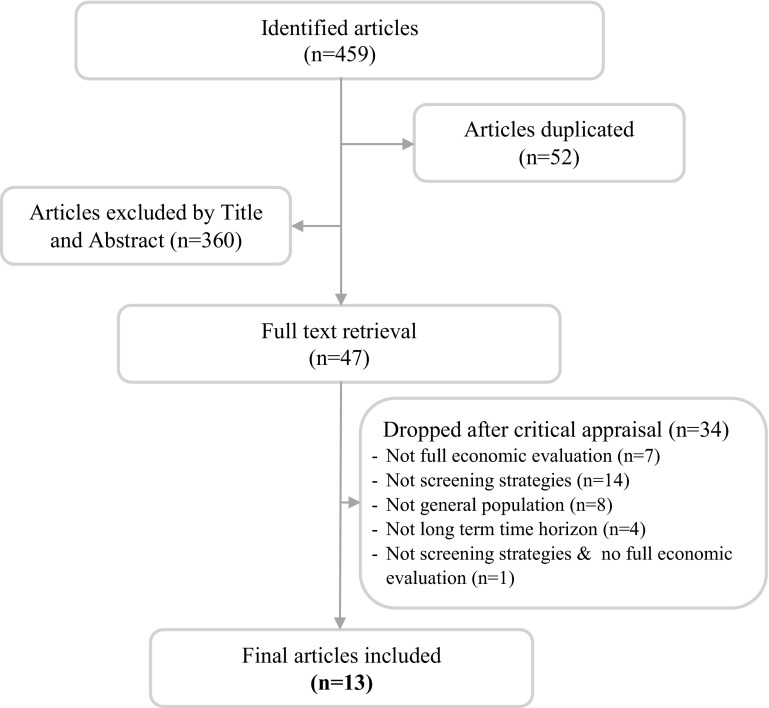
Flow chart for the selection of economic evaluation studies

The literature search initially identified 459 articles, of which 47 were selected for full text retrieval based on relevance of title and abstract (Fig. 1). After reading the full text, 13 articles met the inclusion criteria specified above. The main reasons for excluding the remaining 34 articles were that they did not evaluate screening strategies (*n* = 14), did not involve full economic evaluations (*n* = 7), did not evaluate screening strategies and did not involve full economic evaluation (*n* = 1), were not based in the adult general population (*n* = 8), or had a time horizon <1 year (*n* = 4). Table 1 and eTable 2 summarize the main characteristics of the included studies [18–30].

**Table 1:** Summary of the characteristics of the included studies

Articles	Year	Region/country	Target population/result by subgroup	Strategies of screening compared (S1, S2, S3, etc.)	Treatment	Model	Time horizon	Perspective	Outcome
Blake et al. [18]	2003	US	People aged 35 to 85 years without hyperlipidemia (LDL cholesterol <149 mg/dl)/age and sex	S1: no screening and no treatment (usual care); S2: C-reactive protein screening and treatment; S3: no screening and treat all	Statin	State transition	10 years	Health care	Cost per QALY
Johannesson [19]	2001	Sweden	People aged ≥35 years/sex and age	Screening at different risk thresholds	Statin	State transition	Lifetime	Social	Cost per QALY
Marshall and Rouse [20]	2002	UK	People aged 30 to 74 years/no analysis by subgroup	S1: clinical risk assessment for all patients at age 30; S2: pre-selection of patients for assessment using a prior estimate of their CVD risk that include age, sex, diabetes status, and default values for other risk factors	Aspirin, statin and anti-hypertensives	5-year probability of CVD	5 years	Primary health care	Cost per CVD event prevented
Pletcher et al. [21]	2009	US	People aged 35 to 85 years/age, sex and risk level	S1: Adult Treatment Panel III guidelines; S2: range of risk- and age-based alternative strategies	Statin	State transition	30 years	Health care	Cost per QALY
Kok et al. [22]	2009	The Netherlands	People aged >30 years/age and sex	S1: old guideline S2: new guideline (SCORE)	Statin and anti-hypertensives	State transition	20 years	Health care	Cost per LY; cost per QALY
Rapsomaniki et al. [23]	2011	North America, Western Europe, and Japan	People aged ≥40 years/no analysis by subgroup	S1: gender, region, age and year of birth; S2: additionally includes three established CVD risk factors: SBP, total cholesterol, and smoking status	Statin	Partitioned survival curve	10 years	Health care	Cost per CVD-free year of life
Wald et al. [24]	2011	UK	People aged 0 to 89 years/age and CVD risk cut-off	S1: age alone; S2: FRS	Statin and anti-hypertensives	Individual patient simulation	Lifetime	Health care	Cost per CVD-free year of life
Choudhry et al. [25]	2011	US	Men aged ≥50 years and women ≥60 years with LDL cholesterol <130 mg/dl/no analysis by subgroup	S1: testing hs-CRP and rosuvastatin for patients with hs-CRP ≥2 mg/l; S2: no screening and no treatment (usual care);	Statin	State transition	Lifetime	Social	Cost per QALY
Lovibond et al. [26]	2011	UK	People aged ≥40 years/age and sex	S1: BP monitoring in the clinic (measurements at monthly intervals over 3 months); S2: BP monitoring in the home (measurements over a week); S3: ambulatory monitoring (measurements over 24 h)	Anti-hypertensives treatment	State transition	Lifetime	Health care	Cost per QALY
Cobiac et al. [27]	2012	Australia	People aged ≥35 years/absolute risk and sex	S1: usual care; S2: single risk factor-based guidelines; S3: absolute risk-based guidelines	Statin and anti-hypertensives	State transition	Lifetime	Health care	Cost per QALY
Shiffman et al. [28]	2012	US	People aged 45 to 79 years/sex	S1: FRS; S2: FRS + lipoprotein(a)	Aspirin	State transition	10 years	Health care	Cost per CVD event prevented; cost per QALY
den Ruitjer et al. [29]	2013	US	People aged 50–59 years/sex	S1: FRS; S2. FRS + carotid intima-media thickness	Statin, anti-hypertensives and platelet aggregation inhibitor	State transition	10, 20, and 30 years	Health care	Cost per QALY
Lee et al. [30]	2010	US	People aged ≥40 years/age, sex, and absolute risk	S1: Adult Treatment Panel III guidelines; S2: hs-CRP in those without an indication for statin followed by targeted statin for patients with elevated hs-CRP levels; S3: statin therapy at specified predicted risk thresholds without hs-CRP	Statin	State transition	Lifetime	Health care perspective	Cost per QALY

*CVD* cardiovascular disease, *FRS* Framingham Risk Score, *hs*-*CRP* high-sensitivity C-reactive protein, *LY* life-year, *QALY* quality-adjusted life-year, *S* screening strategy, *SBP* systolic blood pressure

## Critique of the health-economic approaches used by the included studies

### Question 1: What are the criteria for cost-effectiveness?

The quality-adjusted life-year (QALY) was the most commonly used health outcome, measured over the patient’s lifetime or restricted to 10 years. The QALY is a composite measure calculated as the product of survival and health-related quality of life, and is therefore appropriate for a condition such as CVD which impacts on both dimensions of health. Use of alternative metrics such as the number of CVD events prevented or CVD-free life-years gained does not take account of the patient experience after the CVD event.

While the QALY captures both morbidity and mortality, it has been criticized for excluding other considerations that might be important to decision-makers, for example, the effect of the programme on health-related inequalities or vulnerable groups [31], the impact on labor productivity [19], moral hazard (e.g., statins may give a false sense of health security to treated individuals, counteracting the incentive to adopt lifestyle changes), and medicalizing a generally healthy population [32].

Any health gained by implementing a new programme has an opportunity cost of health (and other goods) foregone elsewhere. Some studies used the threshold approach, comparing the incremental cost-effectiveness ratio (ICER) of the intervention with the national threshold set by relevant health-care authorities (Table 1). A fixed ICER threshold may not be appropriate for making decisions about large-scale public health programmes such as national screening if financing these gross changes would successively cut into more essential and productive health services elsewhere. An alternative way to estimate the opportunity cost of introducing a new screening programme is to use the “fixed-budget” method, in which the additional number of individuals treated is fixed up front (e.g., top quartile of the population at greatest CVD risk) and then the strategy that maximizes total health given the fixed budget is considered as the optimal screening strategy [23]. One study [20] calculated an efficiency frontier [33]. This allows dominated options (those at higher cost but no more effective) to be identified and excluded, but unless the decision-maker is willing to specify a cost-effectiveness threshold, does not offer any guidance about choosing between options on the frontier.

### Question 2: What is the structure of the economic model?

The structure of a model represents the important events or “states” whose occurrence or “state-occupancy” are to be predicted. As CVD is a chronic condition, the model should predict events over the full lifetime of the cohort of patients. Decision models can facilitate extrapolation (prediction of events beyond the time horizon of the primary studies), synthesis (bringing together evidence from different and diverse sources), and sensitivity analysis (prediction or simulation under alternative assumptions or data).

The models reviewed were implemented with varying degrees of complexity with between 2 and 33 states (see Table 2 and eTable 3 for a description of the health states in each model). Simpler structures included states such as “no CVD”, “non-fatal CVD event”, and “dead”. Other models distinguished between types of non-fatal CVD events (e.g., stroke, myocardial infarction (MI)), causes of death (e.g., CVD-related, other causes), or included adverse events of treatment as separate health states. More complex models included successive non-fatal CVD events (e.g., stroke followed by MI) or time-dependency (e.g., a tunnel state in a state-transition model to incorporate a higher rate of death in the first year after a non-fatal CVD event, compared to subsequent years after the event). The authors of each study rarely justified why they chose the given model structure and neither did they acknowledge that alternative structures could be implemented. While additional states may allow greater accuracy to predict outcomes, it may be difficult to reliably estimate all the necessary parameters in a complex model. This gives rise to a trade-off between desirable model structure and reliable parameter estimation [34]. Even large epidemiological datasets may not have sufficient observations to give precise estimates of all the transitions in a complex model. Such modeling may produce unreliable results, and so validation is an essential part of the model-building process [35].

**Table 2:** Health states included in the different models

Number of health states	Number of studies	References	Non-fatal health states	Causes of death
2	1	Marshall et al. [20]	Alive without CHD; Alive after CHD	No fatal state
3	1	Johannesson [19]	Alive without CHD; Alive after CHD	Death
4	2	Rapsomaniki et al. [23] and Wald et al. [24]	Alive without CVD; Alive after CVD	CVD; OCM
6	2	Shiffman et al. [28], Lee et al. [30]	Alive without CVD; Alive after MI; Alive after stroke	MI; Stroke; OCM
6	1	Cobiac et al. [27]	Alive without CHD; Alive after CHD; Alive after stroke	Stroke; CHD; OCM
8	1	Kok et al. [22]	Alive without CVD; Alive after MI; Alive after stroke; Alive after other CHD	MI; Stroke; CHD; OCM
8	1	Blake et al. [18]	Alive without CVD; Alive after MI; Alive after stroke; Alive after MI after stroke; Alive after stroke after MI	MI; Stroke; OCM
11	1	Den Ruitjer et al. [29]	Alive without CVD; Alive after first MI; Alive after second MI; Alive after stroke; Alive after hemorrhagic stroke; Alive after gastrointestinal bleeding	MI; Stroke; Hemorrhagic stroke; Gastrointestinal bleeding; OCM
11	1	Pletcher et al. [21]	Alive without CVD; Alive after MI; Alive after stroke; Alive after SA; Alive after MI after SA; Alive after stroke after MI; Alive after revascularization after SA	MI; Stroke; SA; OCM
12	1	Lovibond et al. [26]	Alive without CVD; Alive after MI; Alive after stroke; Alive after UA; Alive after SA; Alive after TIA	MI; UA; SA; Stroke; TIA; OCM
33	1	Choudhry et al. [25]	States are combination of CVD events and complications, diabetes onset, myopathy, and VTE	MI; UA; Stroke; VTE; OCM

*CHD* coronary heart disease, *CVD* cardiovascular disease, *MI* myocardial infarction, *OCM* other cause mortality, *SA* stable angina, *TIA* transient ischemic attack, *UA* unstable angina, *VTE* venous thromboembolism

### Question 3: What are the population and strategies of interest?

A summary of the population and strategies evaluated in each article is shown in eTable 2. Age is a risk factor for both CVD and competing non-vascular causes of death. Of the 13 studies, seven stratified the population by age [18, 19, 21, 22, 24, 26, 30] while the others estimated an average result across all age groups. A concern arises when comparing screening strategies based on risk scoring systems that include age as a risk factor for CVD, that age by itself is a strong non-modifiable risk factor, and therefore a strategy that treats patients above a fixed threshold of absolute risk will predominantly select older people. Risk scores such as the Framingham risk score (FRS) may assign the same absolute 10-year CVD risk to a young person with, say, multiple modifiable risk factors such as high cholesterol and hypertension, as an otherwise healthy older person with no modifiable risk factors [36]. Also, the absolute risk of CVD predicted from scores with age as a risk factor can be misleading as they do not take into account competing risks (i.e., the 10-year CVD risk is calculated “as if” other causes of death do not occur) and are therefore likely to over-estimate the true cumulative probability of CVD especially for older people. Stratifying the population into age groups, and evaluating the model separately for each of them, may increase the efficiency of a screening programme by assigning a different strategy to each age group. For example, Johannesson [19] uses the model to estimate an optimum 10-year risk cutoff for starting statins that increases with age (eTable 2).

Some authors evaluated a sequential screening strategy to try to better discriminate between those people who would benefit from statin therapy and those who would not. Den Ruijter et al. [29] used FRS to classify people into low, medium, and high risk and then used carotid intima-media thickness to reclassify people in the intermediate- and high-risk groups. Marshall and Rouse [20] used age, sex, and other variables routinely held in primary care databases to prioritize patients who were to be invited to a full risk assessment, and Lee et al. [30] used FRS to classify people into low and high risk and then considered C-reactive protein (CRP) screening only in those without an indication for statin followed by targeted statin for patients with elevated CRP levels.

### Question 4: How are primary CVD outcomes defined and assessed?

CVD includes coronary and cerebrovascular events, but the exact definition used varied between studies, making comparison difficult. There are at least three key considerations: (1) whether the study included only coronary events, only cerebral events or both; (2) whether the study included only “hard” outcomes (easily measured reliably and objectively) such as confirmed MI and stroke, or both hard and “soft” outcomes such as unconfirmed MI, revascularization, angina, and transient ischemic attack (TIA); (3) whether the study measured the time to first event as a composite outcome, or the times to each component of CVD as separate events (Table 2 and eTable 4). Two models [19, 21] included only coronary heart disease (CHD) outcomes, which is likely to underestimate the benefits of CVD screening. Three models [22, 25, 26] included both hard and soft CVD outcomes. The remaining studies employed a composite CVD outcome as the first event.

A state transition model requires as inputs estimates of the absolute probabilities of incident CVD outcomes over an appropriate time horizon (typically annual transition probabilities). Typically these will increase with age. Broadly three approaches were used in the articles for estimating these parameters. The first was to calculate the CVD probability using a published risk algorithm. For example, Wald et al. [24] simulated idealized risk factor distributions based on the Health Survey for England 2003 and the population structure of England, and then predicted CVD events based on annual transition probabilities calculated from the FRS algorithm [37]. One major drawback in estimating transition probabilities (or events) from a published prediction model is that it assumes the published model is accurately calibrated for the population under consideration, which may seldom be true [38]. As such, appropriate calibration of the prediction model should first be assured when considering this modeling approach.

The second method was to estimate the annual risks by age or age group directly from individual-level epidemiological data using study duration-as-timescale [39, 40]. For example, Pletcher et al. [21] used a previously published model (CHD Policy model [41]), which was parameterized using estimated age- and sex-specific CHD risk based on logistic regression models fitted to longitudinal data from the Framingham Heart Study over 30 years. Under this approach, estimates of long-term rates of events require long follow-up on large numbers of individuals and may be unreliable due to dropout from the primary study. Furthermore, parametric assumptions are needed to extrapolate beyond the longitudinal data.

The third approach was to estimate risks from individual epidemiological data using age-as-timescale [39, 40]. Risks are estimated for the youngest individual in dataset and as that person ages. Older individuals start to contribute to the risk estimation at their corresponding age at entry into the study, giving rise to left-censored data. This approach has some advantages over the study duration-as-timescale, since it encompasses both the duration of the follow-up and the range of ages of study participants to allow risks to be estimated over a wide age range without resort to parametric assumptions for extrapolation. It estimates risks according to age rather than time in the study, which is appropriate because the point at which participants enter an epidemiological cohort study is usually rather arbitrary and does not correspond with any specific event (such as a diagnosis).

Most studies used a large health survey dataset to represent the distribution of baseline risk factors in the population, such as the Health Survey for England or the National Health and Nutrition Examination Survey in the US. One study used a hypothetical cohort assigned average levels of risk factors assembled from diverse sources [30]. This approach ignores correlations between risk variables, although Wald et al. [24] found that these correlations are low, given age and sex.

### Question 5: How are high risk individuals identified and treated?

Risk scoring systems can be based on individual risk variables (such as age alone, or cholesterol level alone) or based on a continuous score calculated as a weighted sum of multiple variables and expressed as a probability (e.g., FRS). In each case, the strategies evaluated in the screening studies might compare different risk scoring systems (each with a predefined cut-off for identifying high risk individuals), or might aim to find the “optimal” risk cut-off using a single risk scoring system [19, 24, 27] (Table 3; eTable 5).

**Table 3:** Examples of strategies according to type of risk score and cut-off

	Type of risk score	
	Individual risk variable	Composite risk score
Comparisons		
Compares different risk score systems	C-reactive protein screening, where the cut-off for high risk is set at >0.16 mg/dl versus no screening	FRS versus FRS plus an additional risk variable (CIMT), with cut-off in each case when the 10-year CVD risk exceeds 20 %
Compares different cut-offs along the same risk score system	Age >45 years versus age >55 years	Compare cut-offs of FRS 10-year CVD risk of 5, 10, and 15 %

*CIMT* carotid intima-media thickness, *FRS* Framingham Risk Score

For a given distribution of risk scores in the population, and assuming that higher scores correspond to greater probability of the event, decreasing the cut-off for a positive test result will increase the sensitivity (true positive rate) of the test, treating more individuals and potentially preventing more CVD events; but it will also reduce the specificity (increase the false positive rate), resulting in more unnecessary treatment and adverse events. The optimal cut-off might be defined as the point where marginal benefits equal marginal costs [23]. This optimal cut-off may be found by comparing different cut-off points using the model and selecting that with the most favorable ICER (if the ICER is the chosen metric for evaluating efficiency). For an example of this approach, see Wald et al. [24].

All screening studies evaluated pharmacological treatment, and most used statins as the preferred treatment for people at high risk, in some cases alongside other treatments (anti-hypertensive, aspirin, and platelet aggregation inhibitors). Three articles evaluated statins, aspirin, or anti-hypertensive treatments as separate options [20, 26, 28]. Surprisingly, no studies in the review evaluated non-pharmacological interventions such as counseling for lifestyle change.

There are several key questions to address in order to quantify the long-term health-economic benefit of risk reduction, including the magnitude of the treatment effect, its duration, variables that moderate it, and the impact of adverse events and discontinuation (eTable 6). Particular issues in the reviewed studies included the following:

1. Some studies used a treatment effect estimate based on a single randomized controlled trial (RCT) [18, 19]. Guidelines for economic analysis recommend that all relevant evidence is considered, indicating that a meta-analysis is generally preferred [42]. However, there may sometimes be important differences between RCTs that would argue against combining their results.
2. Studies that compared different screening methods in primary prevention mostly considered statins as a class, estimating an average relative risk across multiple types and doses. Pletcher et al. [21] took account of the relationship between statin dose and degree of relative risk reduction, although safety may be a concern with higher doses.
3. Most studies estimated an average treatment effect (relative risk) for all CVD outcomes. A few estimated a distinct treatment effect for each type of outcome (e.g., MI, stroke) [20, 24, 27].
4. No study modeled different relative risks from statins across age and sex subgroups in the main analysis. As sensitivity analyses, Johannesson [19] considered different treatment effects by age and Cobiac et al. [27] modeled different treatment effects for men and women.
5. Patients do not comply with drug therapy for a variety of reasons, including adverse events, intolerance, lack of efficacy, and personal preferences. Some patients will switch to other statins. Those that discontinue therapy completely will no longer incur a cost of treatment and will no longer benefit. Other non-compliant patients might continue to be prescribed statins, and incur a cost, but not benefit from them. An intention-to-treat (ITT) analysis of an RCT will already account for the impact of non-compliance observed in the trial in the measure of relative risk. If the rate of non-compliance in clinical practice differs from that of the RCT, then the ITT estimate of relative risk will be inappropriate for that setting. For example, den Ruijter et al. [29] thought that RCTs would underestimate non-adherence rates seen in practice, and in consequence in the economic model the treatment effect of statins was weakened compared with that estimated by the RCT (i.e., made closer to one).
6. Most studies considered adverse events associated with statins to be rare and to have only short-term consequences or lead to discontinuation. Some studies included longer-term consequences by including a health state of myopathy [21, 25] or diabetes [30]. However, reliable estimation of the incidence rate of a rare event is always a challenge.
7. RCTs comparing statin to no statin have a follow-up of around 2–6 years. Therefore the treatment effect over the longer term is uncertain. Most studies assumed the treatment effect of statin was constant over time while patients remained on drug. Some studies modeled a truncated time horizon (e.g., 5 or 10 years), which assumes that events and deaths occurring after this time are not influential or occur at the same rate in all screening options. Wald et al. [24] and Choudhry et al. [25] assumed the treatment effect tapers off over time.

### Question 6: What resource use, costs, and HRQOL are taken into account?

The majority of the studies took a health care perspective (eTable 7). The health care cost (direct cost) includes the screening costs (inviting, testing and communication of results to the target population), acute clinical CVD events (hospitalization, interventions, procedures, medication), long-term health and social care maintenance incurred in the years after the first CVD event (which may include average costs of subsequent CVD events), and monitoring costs associated with primary care follow-up of those patients identified as high risk for CVD. However, not all studies included each of these costs. For example, Rapsomaniki et al. [23] and Pletcher et al. [21] did not include the screening costs. Other models did not include the CVD event costs [22–24] or the monitoring costs [28].

Two studies took a broader societal perspective. Johannesson [19] estimated loss of productivity due to coronary events, and traveling and time costs for patient screening and treatments. Choudhry et al. [25] included the value of time for patients and informal (unpaid) carers using average hourly wages of age-matched US workers. Prevention strategies will reduce the incidence of CVD, and so will directly increase population health. The programme may also make workers more productive, and so will indirectly generate wider social benefits for other sectors of society. However, prevention strategies may also impose a greater cost on the health service, displacing other health care programmes, and in this case will generate an opportunity cost in loss of health and loss of wider social benefits elsewhere. If one takes a broader societal perspective by including the impact on labor productivity of the new programme in the cost-effectiveness ratio, then decision-makers should also consider what is the value of these displaced social benefits, alongside the value of displaced QALYs to the health service (the cost-effectiveness threshold) [43].

Most models [18, 19, 21–24, 26–29] used a fixed price for statins throughout the model time horizon, estimated either by the price of standard doses of a specific statin [19, 24], or by averaging the annual cost of a group of statins [18, 27, 29], or as the lowest price on the market [21]. One study lowered the price over time to take into account foreseeable patent expiry and the expected competition offered by generics [25]. However, if one is to take account in these models of plausible long-term market conditions that have not yet been realized, then one might also need to take account of possible innovations in pharmaceuticals in the product pipeline which may both increase the effectiveness of primary prevention and the cost.

There were considerable differences between studies in the estimated impact on health-related quality of life (HRQOL). Three articles did not take account of HRQOL. For the HRQOL of individuals without CVD, three studies used age and sex-adjusted values from the general population [18, 26, 29], one study used only age-adjusted utilities [19], and another used only sex-adjusted utility [28]. The others used constant utility values ranging from 0.85 to 1. Four studies included disutility from adverse events of statins [21, 25, 28, 30], and only one considered disutility arising from taking medication every day: Choudhry et al. [25] included, in a sensitivity analysis, a reduction of utility of 0.02 per year.

### Question 7: How is the model implemented and validated?

The models were implemented as survival curves, individual patient simulation (IPS) or as state-transition models (Table 1). The survival curve approach used by Rapsomaniki et al. [23] calculated the 10-year probability of CVD-free survival from epidemiological cohorts, and estimated 10-year CVD event-free life-years directly as the area under this curve. Marshall and Rouse [20] assumed that the 10-year percentage probability of CVD calculated using the FRS can be interpreted as the number of CVD events that would be expected to occur within 10 years per 100 patients. However, this is an over-estimate as it fails to account for other causes of death [44].

The IPS models (also known as Discrete Event Simulation or micro-simulation) predict specific outcomes for each individual in a large cohort, each of whom is assigned a particular set of baseline characteristics and passes through the model one at a time. Risk equations govern the probability of events. The model records events and the time until the event for the same individual with screening (and treatment) and without screening until death. The output of the model is then the distribution of outcomes with and without the screening. The remaining studies in this review were implemented as state-transition models, estimating the proportion of the original cohort that is in each of the model states at the end of each discrete time period “cycle”. State-transition models are often limited to simpler structures than IPS models. The transition probabilities are calculated for the cohort as a whole, or for a particular set of baseline characteristics, whereas the transition probabilities in an IPS can be calculated from simulated baseline characteristics of each individual and can depend on the history and timing of events that occur during the model.

A perceived advantage of an IPS model over a state-transition model is that it allows more complexity to be simulated at the individual level, including interactions between intermediate variables (such as cholesterol level) and final outcomes (such as CVD). However, the validity of IPS models depends on having good-quality data to generate the participant-level characteristics and specifying the transition rates for that individual, which may not always be available [34]. An advantage of state-transition models is that they are usually faster to calculate, because they have fewer states and because they do not predict lifetime histories for every individual. This is particularly important for calculating uncertainty in the predictions using probabilistic sensitivity analysis [45].

A central purpose of a model is to provide unbiased and reliable predictions. Hence validation is of great importance [46]. In the context of a decision model, internal validity focuses on the appropriateness of methods used to construct the model and obtain the data inputs. The statistical method for estimating model parameters from the primary data should address overfitting, for example, by cross-validation. The appropriateness of the statistical method was not discussed in any of the reviewed papers.

External validity compares model predictions with observed data in the target population, which may differ somewhat from the data used to construct the input parameters [45]. Wald et al. [24] compared the expected performance of age screening based on the expected age-specific incidence of CVD events using the FRS algorithm with those observed from CVD registry data. Pletcher et al. [21] calibrated the model to reproduce national data on risk factor distributions and CHD outcomes (eTable 8).

Sensitivity analysis tests the robustness of the results to changes in the inputs or structure. This can be used to check that the model responds in the anticipated direction to changes in the inputs. It is also used as a method of testing the responsiveness of the decision model to plausible variation in input values. All studies conducted one-way sensitivity analysis, that is, changing one input leaving others unchanged. Some conducted two-way sensitivity analysis, for example, calculating the ICER for each screening option at different levels of screening cost and cost of preventative treatment [24]. No studies tested alternative model structures (to address structural uncertainty). Some studies conducted probabilistic sensitivity analysis (PSA) for estimating confidence intervals around predictions of costs and QALYs and the overall probability that screening is cost-effective. PSA is implemented by Monte Carlo simulation to jointly sample from all the uncertain parameter distributions. No study took account of possible correlations between parameters when implementing PSA [47].

## Discussion and tentative recommendations for good practice

The construction of a decision model requires choices about a series of interrelated questions regarding the population, intervention, outcomes, the definition of high CVD risk, validation, and the criteria for cost-effectiveness. In this section, we pull together the findings of the literature review and offer some tentative recommendations for good practice for modeling, or, at least, identify weak modeling methods that could lead to misleading results.

Because risk algorithms such as FRS do not take account of competing risks, their calculations of absolute 10-year risk are an overestimate of the probability of CVD (as represented by the cumulative incidence). The FRS algorithm might be hence best used as an instrument to rank people in relative order of priority for primary prevention treatment, rather than as a reliable estimate of the actual probability of CVD in the model. Annual risks of CVD and other events should be calculated from a longitudinal dataset using credible econometric methods. As attrition due to loss to follow up is likely to be a problem in longitudinal studies, an attractive alternative method in datasets where follow-up is relatively short but the distribution of ages is relatively wide may be to estimate risks of events using age as timescale rather than study duration as timescale. As CVD is a chronic disease, a lifetime model horizon is preferable, therefore some degree of extrapolation may be unavoidable. The distribution of baseline risks should be estimated from representative large-scale population-based cohorts or health surveys to capture correlations between risk variables.

Estimation of disease-free survival, overall survival, and QALYs requires a multi-state model structure that links non-fatal and fatal outcomes. The design and implementation of this structure and the outcomes modeled depends to some extent on the purpose of the study and the data at hand, but one should be aware that more complex structures do not always provide more reliable or accurate predictions.

Programmes that screen the whole population by fixed cut-off (such as treating all persons with 10-year risk >20 %) will identify predominantly elderly people, who may not be those most likely to benefit from long-term CVD prevention. The optimal risk cut-off for implementing primary prevention may need to vary by age. More attention should also be directed to evaluation of sequential screening, with the aim of targeting scarce resources where they are most likely to benefit.

The definition of CVD varied considerably between studies. A model that only considers CHD and not cerebrovascular outcomes is likely to underestimate the benefits of screening. Including both hard and soft CVD outcomes can create difficulties in parameter estimation and requires a complex model structure. The definition of CVD in the model should be consistent with that of the algorithm used to predict individual CVD risk.

All studies in this review evaluated statins, or combinations of pharmacological interventions. Implementing general screening for CVD risk would medicalize a wide segment of the general population. Relatively little attention has been given to the potential risks of this strategy, such as adverse events, duration of effectiveness beyond the primary study period, non-compliance and over-medicalizing. More research is also needed on the potential benefits and costs of non-pharmacological interventions, either as complements or, possibly, substitutes for drug therapy. The estimation of relative risk of interventions should be taken from meta-analysis of RCTs (rather than single trials) where possible, but attention needs to be paid to whether the RCTs reflect outcomes achievable in practice.

Validation of models is critical and needs to be improved. Recommendations for good practice include: conduct sensitivity analysis to alternative parameters, test alternative model structures, and use unbiased, efficient and robust statistical methods to estimate parameters from primary data. Validation of parameter estimation might include cross-validation, external validation against data sources not used to build the model, and re-calibration of risk-score equations to the target population.

Confidence intervals for predictions have traditionally been estimated in economic evaluations by probabilistic sensitivity analysis, but this only takes account of parameter uncertainty and not structural model uncertainty. Implementation of PSA should ideally take account of correlations between parameters.

The conventional criterion for cost-effectiveness is the cost-per-QALY threshold. This has been successfully applied in health technology assessment for many years, but may be unsuitable for large scale public health interventions with a substantial budget impact. Alternative approaches might assume a fixed overall budget, or assume a fixed number of persons will be treated. Given the substantial impact of CVD on the wider economy, a societal perspective may be justified, but in this case an evaluation should also take account of the productivity that will be lost by displaced health programmes.

In this literature review, we were primarily interested in identifying the approaches used to model costs and long-term health benefits of CVD risk assessment in the general population. Identifying the methodological issues and the solutions proposed in the literature was considered more important than completeness. Nevertheless we believe our review successfully identified the main issues and approaches.

## Electronic supplementary material

Below is the link to the electronic supplementary material.

Supplementary material 1 (DOC 224 kb)

## References

[1] Leal J, Luengo-Fernandez R, Gray A, Petersen S, Rayner M (2006). Economic burden of cardiovascular diseases in the enlarged European Union. Eur. Heart J..

[2] Goff DC, Lloyd-Jones DM, Bennett G, Coady S, D’Agostino RB, Gibbons R (2014). ACC/AHA guideline on the assessment of cardiovascular risk: a report of the American College of Cardiology/American Heart Association Task Force on Practice Guidelines. Circulation.

[3] Stone NJ, Robinson JG, Lichtenstein AH, Bairey Merz CN, Blum CB, Eckel RH (2014). 2013 ACC/AHA guideline on the treatment of blood cholesterol to reduce atherosclerotic cardiovascular risk in adults: a report of the American College of Cardiology/American Heart Association Task Force on Practice Guidelines. Circulation.

[4] Eckel RH, Jakicic JM, Ard JD, de Jesus JM, Houston Miller N, Hubbard VS (2014). 2013 AHA/ACC guideline on lifestyle management to reduce cardiovascular risk: a report of the American College of Cardiology/American Heart Association Task Force on Practice Guidelines. Circulation.

[5] Perk J, De Backer G, Gohlke H, Graham I, Reiner Z, Verschuren M (2012). European Guidelines on cardiovascular disease prevention in clinical practice (version 2012). The fifth joint task force of the European society of cardiology and other societies on cardiovascular disease prevention in clinical practice (constituted by representatives of nine societies and by invited experts). Eur. Heart J..

[6] Public Health England. NHS Health Check: our approach to the evidence. http://www.healthcheck.nhs.uk/document.php?o=346 (2013) Accessed Feb 2014

[7] National Clinical Guideline Centre. Lipid modification: cardiovascular risk assessment and the modification of blood lipids for the primary and secondary prevention of cardiovascular disease. London (UK): National Institute for Health and Care Excellence (NICE), p. 50 (Clinical guideline; no. 181). http://www.nice.org.uk/guidance/cg181/resources/guidance-lipid-modification-cardiovascular-risk-assessment-and-the-modification-of-blood-lipids-for-the-primary-and-secondary-prevention-of-cardiovascular-disease-pdf (2014) Accessed Oct 2014 25340243

[8] Marshall T (2005). Evaluating national guidelines for prevention of cardiovascular disease in primary care. J. Eval. Clin. Pract..

[9] Murray CJ, Lauer JA, Hutubessy RC, Niessen L, Tomijima N, Rodgers A (2003). Effectiveness and costs of interventions to lower systolic blood pressure and cholesterol: a global and regional analysis on reduction of cardiovascular-disease risk. Lancet.

[10] Chamnan P, Simmons RK, Khaw KT, Wareham NJ, Griffin SJ (2012). Estimating the potential population impact of stepwise screening strategies for identifying and treating individuals at high risk of Type 2 diabetes: a modelling study. Diabet. Med..

[11] Abramson, J.D., Rosenberg, H.G., Jewell, N., Wright, J.M.: Should people at low risk of cardiovascular disease take a statin? Br. Med. J. **347**, f6123 (2013) 10.1136/bmj.f6123 24149819

[12] Mihaylova BBA.: The Cost-Effectiveness of Statin Treatment: Can We Synthesize Evidence From Economic Evaluations? Oxford: Health Economics Research Centre, University of Oxford. http://www.herc.ox.ac.uk/conferences-and-presentations/20012002presentationfilelinks/bm1201 (2001). Accessed Jan 2014

[13] Ara R, Basarir H, Ward SE (2012). Principles of health economic evaluations of lipid-lowering strategies. Curr. Opin. Lipidol..

[14] Neyt M, De Laet C, Van Brabandt H, Franco O, Ramaekers D (2009). Cost-effectiveness of statins in the primary prevention of cardiovascular disease: a systematic review and economic analysis for Belgium. Acta Cardiol..

[15] Franco OH, Peeters A, Looman CWN, Bonneux L (2005). Cost effectiveness of statins in coronary heart disease. J. Epidemiol. Community Health.

[16] Mitchell AP, Simpson RJ (2012). Statin cost effectiveness in primary prevention: a systematic review of the recent cost-effectiveness literature in the United States. BMC Res. Notes.

[17] Schwappach DLB, Boluarte TA, Suhrcke M (2007). The economics of primary prevention of cardiovascular disease: a systematic review of economic evaluations. Cost Eff. Resour. Alloc..

[18] Blake GJ, Ridker PM, Kuntz KM (2003). Potential cost-effectiveness of C-reactive protein screening followed by targeted statin therapy for the primary prevention of cardiovascular disease among patients without overt hyperlipidemia. Am. J. Med..

[19] Johannesson M (2001). At what coronary risk level is it cost-effective to initiate cholesterol lowering drug treatment in primary prevention?. Eur. Heart J..

[20] Marshall T, Rouse A (2002). Resource implications and health benefits of primary prevention strategies for cardiovascular disease in people aged 30 to 74: mathematical modelling study. BMJ.

[21] Pletcher MJ, Lazar L, Bibbins-Domingo K, Moran A, Rodondi N, Coxson P (2009). Comparing impact and cost-effectiveness of primary prevention strategies for lipid-lowering. Ann. Intern. Med..

[22] Kok L, Engelfriet P, Jacobs-van der Bruggen M, Hoogenveen RT, Boshuizen HC, Verschuren MW (2009). The cost-effectiveness of implementing a new guideline for cardiovascular risk management in primary care in the Netherlands. Eur. J. Cardiovasc. Prev. Rehabil..

[23] Rapsomaniki E, White IR, Wood AM, Thompson SG (2012). A framework for quantifying net benefits of alternative prognostic models. Stat. Med..

[24] Wald NJ, Simmonds M, Morris JK (2011). Screening for future cardiovascular disease using age alone compared with multiple risk factors and age. PLoS ONE.

[25] Choudhry NK, Patrick AR, Glynn RJ, Avorn J (2011). The cost-effectiveness of C-reactive protein testing and rosuvastatin treatment for patients with normal cholesterol levels. J. Am. Coll. Cardiol..

[26] Lovibond K, Jowett S, Barton P, Caulfield M, Heneghan C, Hobbs FD (2011). Cost-effectiveness of options for the diagnosis of high blood pressure in primary care: a modelling study. Lancet.

[27] Cobiac LJ, Magnus A, Barendregt JJ, Carter R, Vos T (2012). Improving the cost-effectiveness of cardiovascular disease prevention in Australia: a modelling study. BMC Public Health.

[28] Shiffman D, Slawsky K, Fusfeld L, Devlin JJ, Goss TF (2012). Cost-effectiveness model of use of genetic testing as an aid in assessing the likely benefit of aspirin therapy for primary prevention of cardiovascular disease. Clin. Ther..

[29] den Ruijter HM, Vaartjes I, Sutton-Tyrrell K, Bots ML, Koffijberg H (2013). Long-term health benefits and costs of measurement of carotid intima-media thickness in prevention of coronary heart disease. J. Hypertens..

[30] Lee KK, Cipriano LE, Owens DK, Go AS, Hlatky MA (2010). Cost-effectiveness of using high-sensitivity C-reactive protein to identify intermediate- and low-cardiovascular-risk individuals for statin therapy. Circulation.

[31] Paris, V., Belloni, A.: Value in Pharmaceutical pricing. OECD Health Working Paper No. 63 (2013)

[32] Ridker PM, Cook NR (2013). Statins: new American guidelines for prevention of cardiovascular disease. Lancet.

[33] Caro JJ, Nord E, Siebert U (2010). The efficiency frontier approach to economic evaluation of health-care interventions. Health Econ..

[34] Karnon J, Stahl J, Brennan A, Caro JJ, Mar J, Moller J (2012). Modeling using discrete event simulation: a report of the ISPOR-SMDM modeling good research practices task force-4. Med. Decis. Making.

[35] van Kempen BJH, Ferket BS, Hofman A, Spronk S, Steyerberg E, Hunink MGM (2012). Do different methods of modeling statin treatment effectiveness influence the optimal decision?. Med. Decis. Making.

[36] D’Agostino RB, Vasan RS, Pencina MJ, Wolf PA, Cobain M, Massaro JM (2008). General cardiovascular risk profile for use in primary care: the Framingham Heart Study. Circulation.

[37] Anderson K, Odell P, Wilson P, Kannel W (1991). Cardiovascular disease risk profiles. Am. Heart J..

[38] Beswick AD, Brindle P, Fahey T, Ebrahim S (2008). A Systematic Review of Risk Scoring Methods and Clinical Decision Aids Used in the Primary Prevention of Coronary Heart Disease (Supplement) [Internet].

[39] Korn EL, Graubard BI, Midthune D (1997). Time-to-event analysis of longitudinal follow-up of a survey: choice of the time-scale. Am. J. Epidemiol..

[40] Collett D (2003). Modelling Survival Data in Medical Research.

[41] Mcneil JJ, Peeters A, Liew D, Lim S, Vos T (2001). A model for predicting the future incidence of coronary heart disease within percentiles of coronary heart disease risk. J. Cardiovasc. Risk.

[42] Caro JJ, Briggs AH, Siebert U, Kuntz KM (2012). Modeling good research practices-overview: a report of the ISPOR-SMDM modeling good research practices task force-1. Value Health.

[43] Claxton, K., Walker, W., Palmer, S., Sculpher, M.: Appropriate Perspectives for Health Care Decisions. CHE Research Paper 54. Centre for Health Economics, University of York (2010)

[44] Putter H, Fiocco M, Geskus RB (2007). Tutorial in biostatistics: competing risks and multi-state models. Stat. Med..

[45] Brennan A, Akehurst R (2000). Modelling in health economic evaluation: What is its place? What is its value?. Pharmacoeconomics.

[46] Spiegelhalter DJ, Best NG, Carlin BR, van der Linde A (2002). Bayesian measures of model complexity and fit. J. R. Stat. Soc. Ser. B Stat. Methodol..

[47] Briggs AH, Weinstein MC, Fenwick EAL, Karnon J, Sculpher MJ, Paltiel AD (2012). Model parameter estimation and uncertainty analysis: a report of the ISPOR-SMDM Modeling good research practices task force working group-6. Med. Decis. Making.

