# Supplementary material for: Modeling the costs and long-term health benefits of screening the general population for risks of cardiovascular disease: a review of methods used in the literature
Source: Eur J Health Econ. 2015 Dec 18;17(8):1041–53. doi: 10.1007/s10198-015-0753-2 (PMC5047941; doi:10.1007/s10198-015-0753-2)
Supplement: Supplementary file 1 — Supplementary material 1 (DOC 224 kb) [file 10198_2015_753_MOESM1_ESM.doc]

**Modelling the costs and long-term health benefits of screening the general population for risks of cardiovascular disease: a review of methods used in the literature**

**Supplementary materials**

[eTable 1. Search strategy for Medline and Web of Knowledge 2](#__RefHeading___Toc429588891)

[eTable 2. Summary of the main characteristics and results of the included studies 3](#__RefHeading___Toc429588892)

[eTable 3. Model design and extrapolation 5](#__RefHeading___Toc429588893)

[eTable 4. Definition of primary CVD outcomes and methods used to estimate distribution of population characteristics and baseline risk of CVD 6](#__RefHeading___Toc429588894)

[eTable 5. Threshold for defining a high risk individual 8](#__RefHeading___Toc429588895)

[eTable 6. Primary prevention of CVD event 10](#__RefHeading___Toc429588896)

[eTable 7. Resource use, Costs and Health Related Quality of Life in the model 12](#__RefHeading___Toc429588897)

[eTable 8. Validation and Sensitivity analyses 14](#__RefHeading___Toc429588898)

eTable 1. Search strategy for Medline and Web of Knowledge

**Search strategy for Medline**

|  |  |  |
| --- | --- | --- |
| **Search** | **Terminology used** | **Result** |
| #1  #2  #3 | *("Costs and Cost Analysis"[Mesh] OR "cost-utility" [TI] OR "Cost-effectiveness"[TI] OR "economic evaluation"[TI]) AND ("Cardiovascular Diseases"[Mesh] OR "Coronary Disease"[Mesh] OR "Hypercholesterolemia"[Mesh])*  Search (#1) *AND ("Primary Health Care"[Mesh] OR "primary prevention"[TI] OR "primary care"[TI])*  Search (#2) *AND (“Mass Screening”[Mesh] or "screening"[TI] or "risk assessment" [TI] or "risk evaluation"[TI] or "risk factor"[TI])* | 9602  822  248 |

**Search strategy for Web of Knowledge**

| **Search** | **Terminology used** | **Result** |
| --- | --- | --- |
| #1    #2  #3 | **Topic:** *("cost analysis "or "cost utility" or "cost benefit" or "cost effectiveness" or “economic evaluation”)* *AND* **Topic:** *("cardiovascular disease" or "coronary" or "Hypercholesterolemia")*  *Search (#1) AND* **Topic:** *("risk factor" or "screening" or "risk assessment" or "risk evaluation" or "risk level")*  *Search (#2) AND* **Topic:** *("primary health care" or "primary prevention" or "primary care")* | 7326  642  211 |

eTable 2. Summary of the main characteristics and results of the included studies

| **Study and year publication** | **Description of strategies compared** | **Setting** | **Target population / subgroups** | **Main results** |
| --- | --- | --- | --- | --- |
| **Rapsomaniki et al 2011 (23)** | Two screening strategies modeled: M1 includes gender, region, age and year of birth; M2: additionally includes 3 established CVD risk factors: SBP, total cholesterol and smoking status. | UK | General population aged 40 and over / no analysis by subgroup | Assuming that the value of one EFLY is £20 000 and that the population of England and Wales aged above 40 has the same risk distribution as that in the ERFC data, using M2 instead of M1 would make a net saving equivalent to 38 836 EFLYs or £776:2 million in the first 10 years. |
| **Wald et al (24)** | Age alone (age screening) versus regular assessment using FRS | UK | General populationaged from 0 to 89 / age and CVD risk cut-off | Age screening for future CVD events is simpler than FRS screening with a similar screening performance and cost-effectiveness. It avoids blood tests and medical examinations. The advantages of age screening in the prevention of heart attack and stroke warrant considering its use in preference to multiple risk factor screening. |
| **Cobiac et al 2012 (27)** | Current practice in Australia (based on self reported use of blood pressure drugs, lipid drugs or both, in a national survey); prevention as intended under the current single risk factor-based guidelines; and prevention according to proposed absolute risk-based guidelines, in comparison to no intervention for prevention of CVD. | Australia | General population aged 35 and older / Absolute risk and sex | CVD prevention based on absolute risk is more cost-effective than prevention under the current guidelines, which are based on single risk factor thresholds, and more cost-effective than the current practice. Recommending blood pressure-lowering drugs to everyone with at least 5% absolute risk and statin drugs to everyone with at least 10% absolute risk, can achieve current levels of population health, while saving $5.4 billion for the Government over the lifetime of the population. |
| **Johannesson 2001 (19)** | To estimate at what coronary risk it is cost-effective to initiate cholesterol lowering drug treatment in primary prevention for men and women of different ages | Sweden | General population / Men and women at eight different ages: 35, 40, 45, 50, 55, 60, 65 and 70 years | The risk cut-off value for when treatment is cost-effective varied with age and gender. If society is willing to pay $60 000 to gain a QALY it was cost-effective to initiate treatment if the 5-year-risk of CHS exceeded 2.4% for 35-year-old men, 4.6% for 50-year-old men, and 10.4% for 70-year-old men. The corresponding risk cut-off values for women were 2.0%, 3.5% and 9.1% |
| **Ruitjer et al 2013 (29)** | Compare FRS versus FRS+CIMT measurement | US | Men and women age 50-59 years)/ Sex | Performing CIMT measurements in asymptomatic men and women aged 50–59 years results in additional, but small, health benefits. It takes time for these health benefits to outweigh the initial CIMT measurement costs. Our results support CIMT measurements for cardiovascular risk stratification, in particular for women, when focusing on long-term health |
| **Pletcher MJ et al 2009 (21)** | ATP III guidelines and compare with a range of risk- and age-based alternative strategies. | US | General population age 35 to 85 yr / subgroups by age, sex and risk level | The ATP III guidelines are relatively cost-effective and would have a large public health impact if implemented fully in the United States. Alternate strategies may be preferred, however, depending on the cost of statins and how much society is willing to pay for better health outcomes. |
| **Blake et al 2003 (18)** | Three strategies: no C-reactive protein screening and no statin therapy (i.e., usual care); C-reactive protein screening followed by targeted statin therapy for patients with elevated C-reactive protein levels; or statin therapy for all patients | US | Selected population: hypothetical cohorts of men and women who had no hyperlipidemia (LDL cholesterol ≤149 mg/dL) / ASge and sex | C-reactive protein screening to target statin therapy for the primary prevention of cardiovascular disease may be relatively cost-effective in middle-aged patients without overt hyperlipidemia. Cost-effectiveness ratios improved with increase in age and were higher for women. If a 58-year-old man who smokes and is hypertensive was considered, screening for C-reactive protein followed by statin therapy would be cost saving if the cost of statin therapy was reduced to $500 per year. |
| **Kok et al 2009 (22)** | SCORE risk function versus old guideline | Netherland | Dutch population age >30 / Age and sex | The implementation of a new guideline would be cost effectiveness up to the age of 70 years. In all age group, the cost effectiveness ratio was more favourable for men than for women. |
| **Shiffman et al 2012 (28)** | FRS versus FRS+ LPA testing | US | US population age 45 to 79 year / Sex | To include LPA testing in the context of the aspirin use guidelines for primary prevention could be cost effectiveness |
| **Choudhry et a 2011 (25)** | Usual care versus testing hs-CRP and rosuvastatin for patient with hs-CRP levles ≥ 2mg/l | US | Men ≥50 and  Women≥ 60 with LDL cholesterol level of <130 mg/dl / no analysis by subgroup | Rosuvastatin is cost effectiveness for patient with LDL <130 mg/dl and hs-RP levels ≥2.0mg |
| **Lovinbond et al 2011 (26)** | Different diagnostic strategies for hypertensión | UK | Population aged ≥40 year / Age and sex | Ambulatory monitoring was the most cost effectiveness strategy for the diagnosis of hypertension for men and women of all ages. |
| **Marshall and Rouse 2002 (20)** | Five yearly assessment for risk of CVD for all patient using FRS versus pre-selection of patients for assessment using a prior estimate of their risk of cardiovascular disease using age, sex, diabetes and default values for other risk factor (using FRS). | UK | Population aged 30 to 74 / no analysis by subgroup | Preselecting patients for clinical assessment of CVD disease is cost effective than assessing all adults. Moreover, treating many patients with low cost drug is more efficient than prescribing a few patients intensive antihypertensive and statins. |
| **Lee et al 2010 (30)** | Three strategies: Adult Treatment Panel III guidelines; hs-CRP screening in those without an indication for statin followed by targeted statin for patients with elevated hs-CRP levels; or statin therapy at specified predicted risk thresholds without hs-CRP testing patients | US | Hypothetical cohorts, starting at 40 years of age, with normal lipid levels and no coronary artery disease, peripheral arterial disease, or diabetes mellitus /subgroup with hypertension / subgroup with hypertension and smoking | Strategy 3 (risk-based treatment without hs-CRP testing) was the most cost-effective, assuming that statins were equally effective regardless of hs-CRP status. However, if normal hs-CRP levels identified a subgroup with little or no benefit from statin therapy (20% relative risk reduction), then Strategy 2 (hs-CRP screening) would be the optimal strategy. If harms from statin use were greater than generally recognized, then Strategy 1 (current clinical guidelines) would be optimal. |

CVD: cardiovascular disease; SBP: systolic blood pressure; FRS: Framingham risk score; CIMT: carotid intima-media thickness; LPA: lipoprotein(a) gene; hs-CRP: high-sensitivity C-reactive protein; EFLY: Event free life year; ERFC: Emerging Risk Factor Collaboration study; ATP: Adult Treatment Panel

eTable 3. Model design and extrapolation

| **Study** | **Model design used** | **Time horizon** | **Discounting rates**  **Cost/outcome** | **Health states or clinical outcomes modelled** | **Final health outcome** | **Threshold CE** |
| --- | --- | --- | --- | --- | --- | --- |
| **Rapsomaniki et al 2011 (23)** | Survival model | 10 year | Not included | No event / non fatal CVD or death | CVD free year of life gained | £20 000 / CVD free year of life gained |
| **Wald et al (24)** | Individual patient simulation | Lifetime | Sensitivity analysis only | No event / CV event | CVD free year of life gained | no specific criteria |
| **Cobiac et al 2012 (27)** | State transition | Lifetime | 3%/3% | No event, non fatal CHD / non fatal stroke / All cause mortality | QALY | AUS$ 50,000/QALY |
| **Johannesson 2001 (19)** | State transition | Lifetime (up to 110 year old) | 3%/3% | No event, Non-fatal CHD / Fatal CHD and Fatal non CHD. | QALY | range of threshold ($40,000, $60000, $100,000) |
| **Ruitjer et al 2013 (29)** | State transition | 10, 20 and 30 years | 3%/3% | No event/fatal or non fatal first MI / fatal or non fatal second MI/ ischemic stroke / hemorrhagic stroke/ gastrointestinal bleeding / OCM | QALY | Range of threshold( US $ 50,000/QALY and US$100,000/QALY. |
| **Blake et al 2003 (18)** | State transition | Lifetime | 3% / 3% | No event / fatal MI / nonfatal MI / fatal stroke / nonfatal stroke / OCM | QALY | no specific criteria |
| **Pletcher MJ et al 2009 (21)** | State transition | 30 years | 3% / 3% | No event, non fatal CHD, subsequent CHD, fatal CHD, other cause of mortality | QALY | $50,000/QALY |
| **Kok et al 2009 (22)** | State transition | 20 years | 4% / 1% | No event / non fatal MI/Non fatal stroke/nonf falta other CHD/all cause mortality | LYG and QALY | €20,000 /QALY |
| **Shiffman et al 2012 (28)** | State transition | 10 years | 3.5% / 0% | No event/ MI /stroke/ GI bleeding | CVD event prevented / QALY | no specific criteria |
| **Choudhry et a 2011 (25)** | State transition | Lifetime | 3% / 3% | No event / fatal and non fatal Stroke/ fatal and non fatal MI / fatal and non fatal UA / fatal and non fatal VTE/ fatal and non fatal revascularization/ fatal and non fatal multiple event/fatal stroke/ Non CV death  Post-ACS /Post VTE /OCM | QALY | $50,000/QALY |
| **Lovinbond et al 2011 (26)** | State transition | Lifetime | 3,5% / 3,5% | No event /Non fatal MI / Post MI /Non fatal UA/Post UA / Non fatal SA /Post SA/ | QALY | £20,000 - £30,000 / QALY |
| **Marshall and Rouse 2002 (20)** | Probability of CVD | 5 years | 6% for cost | No event / CVD event | CVD event prevented | Maximising health benefits within total resources |
| **Lee et al 2010 (30)** | State transition | Lifetime | 3% / 3% | No event /Non fatal MI / Post MI /Non fatal stroke /Post stroke / Fatal CVD / OCM (each survival state with/without statin depending on tolerance and with/without elevated hs-CRP) | QALY | $55,000 / QALY |

CVD: cardiovascular; OCM: other cause of mortality; CHD: coronary heart disease; MI: myocardial infarction; GI: Gastrointestinal; UA: unstable angina; SA: stable angina; VTE: venous thromboembolism; ACS: acute coronary syndrome; LYG: life- years gained; QALY: quality-adjusted life years;

eTable 4. Definition of primary CVD outcomes and methods used to estimate distribution of population characteristics and baseline risk of CVD

| **Study** | **Primary outcome/**  **Definition of CVD** | **Description of datasets for population characteristics and natural history of CVD** |
| --- | --- | --- |
| **Rapsomaniki et al 2011 (23)** | CVD / CHD and stroke | Population characteristics: Meta-analysis of individual participant data from 53 cohort studies in the ERFC. Main analysis was restricted to 171,175 participants aged >40 years without history of diabetes or CVD and not under statin treatment (where known) and had complete information on total cholesterol, blood pressure and smoking status. Studies were grouped into four geographic regions for estimation of baseline hazards: USA and Canada, Northern Europe, Southern Europe and Japan. |
| **Wald et al 2011 (24)** | CVD / CHD death, non fatal myocardial infarction and stroke. | Population characteristics: Health Survey for England. Estimates of the annual risk of CHD death, non-fatal MI and stroke, were calculated for each individual patients using FRS. The 10-year incidence rates were converted into single-year estimates by “fitting a log-linear model” to the published incidence estimates. The overall risk of CVD was taken to be the sum of the risks for CHD death, non-fatal MI and stroke. |
| **Cobiac et al 2012 (27)** | CVD / IHD and stroke | Population characteristics: Ausdiab data set for 1999-2000. Annual risk of CVD was derived from the FRS. They assumed that 5% 5 year-risk is approximately equivalent to a 10% 10-year risk , and a 10% 5 year-risk is approximately equivalent to a 20% 10 year risk. |
| **Johannesson 2001 (19)** | CHD / MI, stable and unstable angina pectoris. | Population characteristics: National Board of Health and Welfare in Sweden. Risk model estimated the average 5 year risk of CVD based on FRS. Unclear how annual probabilities were derived from cumulative risk |
| **Ruitjer et al 2013 (29)** | CVD / MI and stroke | Population characteristics: ARIC study. Annual risk of CVD was derived from the 10-year FRS. Average annual risk MI for Men (Initial risk ≤ 5% ; 5% < Initial risk ≤ 10%; 10% < Initial risk ≤ 20%; Initial risk ≥ 20% )= (0.002; 0.008; 0.016; 0.031). Corresponding data for women = (0.002; 0.008; 0.015; 0.035). |
| **Blake et al 2003 (18)** | CHD / MI and stroke | Population characteristics: AFCAPS/TexCAPS. Annual CHD risk estimated from AFCAPS/TexCAPS. Annual stroke risk estimated from population studies. The econometric method used is not specified. |
| **Pletcher MJ et al 2009 (21)** | LDL Cholesterol / cardiac arrest, MI, or angina | CHD policy model. Population characteristics: National Health and Nutrition Examination Survey, 1999 to 2004, U.S. population. Annual CHD risk is based on logistic regression models estimated using longitudinal Framingham data. Cumulative Percentage of Persons With a First CHD Event by year (1,2,3,4,5)=(1.6%, 3.3%, 5.1%, 7.0%,8.8%) |
| **Kok et al 2009 (22)** | CVD / AMI, other coronary disease, stroke | Population characteristics: national and local disease registries, Statistics Netherland, and several studies in Dutch population. Absolute 10-year CV risk is estimated based on an adapted version of SCORE risk function. This adaptation consists of a calculation of incidence of CVD, instead of CV mortality as in the original score. Moreover, they assumed the SCORE function remains valid for older age groups. |
| **Shiffman et al 2012 (28)** | CVD / MI and stroke | Population characteristics: National Heath and Nutrition Examination Survey. 10-year risk of CVD is estimated using FRS. |
| **Choudhry et a 2011 (25)** | CVD / MI, unstable angina, revascularisations, stroke, venous thromboembolism, diabetes, elevated liver enzymes, and myopathy. | Population characteristics: JUPITER trial. 10-year risk of CVD is estimated using FRS. |
| **Lovinbond et al 2011 (26)** | CVD / CHD and stroke | Population characteristics: Risk factor inputs derived from Health Survey for England 2006. Non CV mortality was based on England and Wales lifetables. Absolute risk is calculated using FRS. |
| **Marshall and Rouse 2002 (20)** | CVD / stroke, CHD, peripherial vascular disease, heart failure. | Population characteristics: 1998 Health Survey of England. Using this data they generated a hypothetical population whose distribution for age, sex and risk factor represents a typical practitioner list. Absolute risk of CVD is calculated by FRS. |
| **Lee et al 2010 (30)** | CVD / MI and stroke | Population characteristics: Hypothetical cohorts. Distribution of hs-CRP levels from National Heath and Nutrition Examination Survey. Median cholesterol levels from Jupiter trial. Assumes cholesterol level is independent of hypertension and smoking. Absolute CVD risk is calculated by FRS (for unknown hs-CRP) and Reynolds Score (for known hs-CRP level) |

CVD: cardiovascular disease; CHD: coronary heart disease; MI: myocardial infarction; FRS: Framingham risk score; ERFC: Emerging risk Factors Collaboration

eTable 5. Threshold for defining a high risk individual

| **Study** | **Risk score used, description of variables and coefficients/** | **Cutoff used initiating treatment** |
| --- | --- | --- |
| **Rapsomaniki et al 2011 (23)** | Two risk scores are used in two different screening models. Model 1 includes gender, region, age and year of birth; Model 2 additionally includes SBP, total cholesterol and smoking status. | 10 year CVD risk > 20% |
| **Wald et al 2011 (24)** | Two approaches:  a) Age  b)10-year FRS | Age cutoff (for a) 45, 65, 75, 85; FRS 10 year absolute risk (for b): 5% 10% 20% |
| **Cobiac et al 2012 (27)** | Two approaches:  a) Risk factors (single and in combination) based on national guidelines;  b) FRS calibrated for the Australian population. | Combinations of risk factor (for a): Diabetes and age > 60 years; Diabetes and total cholesterol > 5.5 mmol/L;Total cholesterol > 6.5 mmol/L and HDL cholesterol < 1 mmol/L; Total cholesterol > 6.5 mmol/L and hypertension; Total cholesterol > 5.5 mmol/L and HDL cholesterol < 1 mmol/L and hypertension; Total cholesterol > 7.5 mmol/L or triglycerides > 4 mmol/L for men aged 35-75 years or postmenopausal women < 75 years; Total cholesterol > 9 mmol/L or triglycerides > 8 mmol/L. FRS 5 year absolute risk (for b): 5% 10% 15% |
| **Johannesson 2001 (19)** | FRS | The optimal cutoff is the objective of the study and is conditioned by WTP and the rest of variables included in the model. |
| **Ruitjer et al 2013 (29)** | Individuals were first classified based on their 10-year risk of developing CHD based on FRS alone. Those who were considered moderate risk (10–20%) or high risk (>20%) were then reclassified after adding CIMT information to FRS. | Results yielded four classifications of 10-year risk of developing CHD: very low risk (<5%), low risk (5–10%), moderate risk (10–20%) and high risk (>20%). Treatment is started in all high-risk individual (>20% risk in 10 years), and in 20% of the individuals in the intermediate risk category (10-20% risk in 10 years). |
| **Blake et al 2003 (18)** | No risk score used. Treatment criteria are based on C-reactive protein levels | Screened patients with C-reactive protein levels 0.16 mg/dL were considered to have elevated levels and were eligible for statin therapy. |
| **Pletcher MJ et al 2009 (21)** | 6 modifiable CHD risk factors: diastolic blood pressure, LDL cholesterol level, HDL cholesterol level, smoking status, diabetes mellitus (yes or no), and statin use (yes or no), as well as by sex and 10-year age range. Coefficients were conditioned on age and sex. | 10-year CHD risk > 20% |
| **Kok et a 2009 (22)** | Adapted version of SCORE risk | 10-year CVD risk ≥ 10%; Within the range of 5-9% risk, treatment is recommended as an option in the presence of additional risk factor. |
| **Shiffman et al 2012 (28)** | FRS and genetic test of the genotypes of 2 PA variants (rs3798220 and rs10455872) | USPSTF threshold: 10-year CVD risk for MEN: age(45 to 59): ≥4%; age(60-69): ≥9%; age(70-79): ≥12% ; 10-year CVD risk for Women: age(55-59): ≥3%; age(60-69): ≥ 8%; age(70-79): ≥11%  Or 10 year CVD risk for men and women below the USPSTF threshold for aspirin use and positive LPA test |
| **Choudhry et a 2011 (25)** | hs-CRP test | Patient with hs-CRP leves ≥ 2 mg/l |
| **Lovinbond et al 2011 (26)** | Age and BP | Age 40 year or older with BP level greater than 140/90 mm Hg |
| **Marshall and Rouse 2002 (20)** | FRS | Different threshold according treatment:  For aspirin: Age>50 and 5 year CH risk >7.5%  For antihypertensive: systolic BP ≥ 160 mm Hg or systolic BP ≥ 140 mm Hg and 5 year CHD risk >7.5%  For statins: total cholesterol to high density lipoprotein cholesterol ratio ≥ 3.5 and 5 year CVD risk >7.5% |
| **Lee et al 2010 (30)** | FRS  hs-CRP test | 10-year CVD risk ≥ 20% or patient with diabetes mellitus;  Patient with hs-CRP level ≥ 2 mg/l |

SBP: systolic blood pressure; HR: hazard ratio; FR: Framingham risk; FRS: Framingham risk score; WTP: willingness to pay; CHD: coronary heart disease; CVD: cardiovascular disease; USPSTF: United States Preventive Services Task Force; LPA: Lipoprotein(a); hs-CRP: high-sensitivity C-reactive protein

eTable 6. Primary prevention of CVD event

| **Study** | **Statin type** | **Other treatments** | **Effect of treatment for statin or combination of drugs** | **Source of data on relative risk reduction/ Years of follow up in the source data** | **Does treatment effect change over time or after the end of the trial?** | **How are adverse events modelled?** | **How are withdrawals and other non-compliance modelled?** |
| --- | --- | --- | --- | --- | --- | --- | --- |
| **Rapsomaniki et al 2011 (23)** | Statins | No other treatments | RR: 0.80 | Metanalysis: Baigent C et al 2005. / Mean follow up was 4.7 year, range (2- 6 years) | Constant effect during the time horizon of the study. | Not included | Same compliance pattern as the clinical trial participants in metanalysis (i.e.no further adjustment of RR) |
| **Wald et al (24)** | Standard dose statin | 3 half-standard dose BP-lowering drugs | Combination of drugs  RR:0.20 (CHD deaths and non fatal MI)  RR:0.30 (stroke event) | Several studies: Wald NJ et al 2003; Law MR 2003a; Law MR 2003b; Law MR et al 2009 / mean follow up not provided | Some attenuation of effect with increasing age is reported in the study but the change in RR is not clearly quantified. | Not included | Not explicitly modelled |
| **Cobiac et al 2012 (27)** | Several statins | BP-lowering drug. | *For statin treatment*:  RR IHD=0.70  RR Stroke=0.81  (Effect of combinations of drugs determined multiplicatively) | Metanalysis: (Brugts JJ et al 2009, Law MR et al 2009 for blood pressure treatment]), / mean follow up of 4.1 year | Constant effect while patient remains on drug | Not included | Discontinuation of 40% of patient at 12 months based on Australian data on discontinuation of statin and blood pressure-lowering drugs. |
| **Johannesson 2001 (19)** | Pravastatina | No other treatments | RR=0.69 for CHD  No effect of statin on stroke | West of Scotland coronary prevention study of primary prevention. (Shepherd J, 1995) / mean follow-up period of 4.9 years | Constant effect during first 5 years. Assumed no treatment thereafter | Not included | Duration of treatment 5 years. Other scenarios not modelled |
| **Ruitjer et al 2013 (29)** | Statins | BP lowering drugs, and a platelet aggregation inhibitor. | Combination of drugs:  Moderate scenario: RR of MI of 0.675  Conservative scenario: RR of MI of 0.80; Optimistic scenario: RR of MI of 0.55 | Hippisley-Coc J 2005, Yusuf S 2002, Wald NJ et al 2003 / around 2 year of treatment | Constant effect while on drug during the time horizon of the study. | Assumed that adverse events would lead to withdrawal with no long term consequences | In practice, 50% of patients are nonadherent. The risk reduction from RCTs is shrunk by half |
| **Blake et al 2003 (18)** | Lovastatin (40 mg) and pravastatin (40 mg). | No other treatments | RR= 55% (patients with LDL cholesterol levels 149 mg/dL and elevated C-reactive protein levels)  RR=0.90 for stroke | Ridker PM 2001 / mean follow-up of 5.2 year | Constant effect during the time horizon of the study. | Not included | (71% adherence during first 5 years). Non-adherence reduces cost. It is not stated whether risk reduction from RCT was also reduced |
| **Pletcher MJ et al 2009 (21)** | Low intensity statin  high intensity | No other treatments | RR=0.73 (Low intensity statin: LDL-C-lowering)  RR=0.45 (%; high intensity statin: LDL-Clowering) | Maron DJ 2000, Kendrach MG 2004, Illingwoth DR 1994 | Constant effect during the time horizon of the study | Myopathy and hepatitis. It was assumed that side effects occurred at twice the rate for high-intensity statins on the basis of data that indicated that some side effects are dose-related. | 100% adherence to primary prevention (base case). 70% and 50% in sensitivity analyses. RR and statin cost are adjusted proportionately. 100% adherence to secondary prevention guidelines in all analyses |
| **Kok L et al 2009 (22)** | Statins | Antihypertensives | Data not provided | Not clearly specified | Not clearly specified | Not included | Not explicitly modelled |
| **Shiffman et al 2012 (28)** | No statin is used | Aspirin | Data not provided | United Stated Preventive Services Task Force guideline | Not clearly specified | Aspirin-related bleeding | They assumed an adherence rate to aspirin use of 80% |
| **Choudhry et a 2011 (25)** | Rosuvastatin | No other treatments | RR: MI: 0.46 ; hospitalization for unstabe angina:0.59; revascularizarion:0.54; stroke:0.52; VTE: 0.57;  RR: diabetes:1.26; elevated liver enzumes:1.24; myopathy:1.11; | JUPITER trial / mean follow up of 1.9 year (maximal follow-up 5.0  years), | Constant effect during first 5 year. Treatment effects persist for 15 years at the level observed during the trial and then tapered off to no effect after 25 years of follow-up. | Model included myopathy as adverse event, and other complications such as diabetes onset and liver function test > 3 times normal. Patient with myopathy or LFT discontinue statin | Not explicitly modelled |
| **Lovinbond et al 2011 (26)** | No statin is used | Anthihypertensive treatment | RR: CHD: 0·633–0·717  Stroke: 0·526–0·717 | Metanalysis : Law MR 2009; mean follow up of 4.1 year | Not clearly specified | No included | Not explicitly modelled |
| **Marshall and Rouse 2002 (20)** | Simvastating 10 mg | Aspirin and anthihypertensive treatment | RR**:** CVD: 0.70  Stroke:0.71 | Metanalysis: LaRosa JC et al 1999. Blauw GJ et al 1997.  Trial: Hebert PR et al 1997/  mean follow up of 5.4 years | Not clearly specified | Model included major bleeding event as adverse event for aspirin treatment | Not explicitly modelled |
| **Lee et al 2010 (30)** | Simvastating 80 mg | No other treatments | RR: MI:0.77;Sroke:0.83 (it assumes that RR is independent of hs-CRP and FR factors)  RR:MI:0.46;Sroke:0.52 (it assumes that RR is not independent of hs-CRP and FR factor | Metanalysis: Baigent C et al 2005. / Mean follow up was 4.7 year, range (2- 6 years)  JUPITER trial / mean follow up of 1.9 year (maximal follow-up 5.0  years), | Not clearly specified | Model included rhabdomyolysis and renal failure for the base case at rate of 1 event per 18,000 patient-year; and a possible increased risk of new-onset DM | 17.5% of individuals will stop statin with 6 months because of intolerance. |

RR: Relative risk BP: blood pressure; CHD: coronary heart disease; MI: myocardial infarction; VTE: venous thromboembolism; CVD: cardiovascular disease; hs-CRP: high sensitivity C-reactive protein; FR: Framingham; DM: diabetes mellitus;

eTable 7. Resource use, Costs and Health Related Quality of Life in the model

| **Study** | **Perspective** | **Costs included (screening and treatment/ CVD event / other costs)** | **Source of data** | **Price year and currency** | **HRQL estimates/ method used for estimation** | **Disutility from taking medicines every day** | **Disutility from statin-related adverse event** |
| --- | --- | --- | --- | --- | --- | --- | --- |
| **Rapsomaniki et al 2011 (23)** | Health care | Screening cost is not included /annual cost of treatment included | Assumption | Year is not provided, UK | Not included | Not included | Not included |
| **Wald et al (24)** | Health care | Screening assessment /Standard doses of statin and other treatment | Assumption | Year is not provided , UK | Not included | Not included | Not included |
| **Cobiac et al 2012 (27)** | Health care | Screening cost: 1 GP visit, 1 blood test. Annual cost of treatment: Two approaches for annual cost of statin: 1. average annual PBS cost for the standard daily dose of statins weighted by scripts provided. 2. Cheapest price of simvastatin (40mg/day), / Cost of other treatments and 1 GP visit and 1 blood test. Costs of treating CVD event are determined separately for the first and subsequent years of treatment. | PBS// Medicare Benefits Schedule costs | 2008, Austalian dollar | Angina:0.904; CHF 0.863; MI: 0.877; Stroke: 0.76 / Quality of Well-being index | Not included | Not included |
| **Johannesson 2001 (19)** | Social perspective | Screening and treatment: laboratory tests, physician visits (include health care cost and travelling and time costs for patient), Statin: 40 mg of pravastatin daily/ CVD events were divided in cost for the first year and annual cost in subsequent year. Include lost productivity (indirect cost) due to coronary event. Future costs were included as the difference between total consumption and production in added life-year. | Previous published studies | 1999 Swedish Crowns (SEK) and convert to US dollars using exchange rates $1=SEK8.50 | Age-adjusted baseline utility from general population. Reduction in HRQL CHD was 0.10 / Time trade-off | Not included | Not included |
| **Ruitjer et al 2013 (29)** | Health care | Screening and treatment: Combination of pharmaceutical treatment; a single additional CIMT measurement. Costs of treating CVD event are determined separately for the first and subsequent years of treatment. | Previous published studies. CIMT measurement cost was based on the commercial price set by University of Chicago Center. | 2011, US dollar | Age and sex-adjusted baseline utility from general population. Multiplicative decrement in utility due to first MI=0.881, second MI=0.800; minor stroke= 0.75; major stroke=0.50; gastrointestinal bleeding= 0.94/ EQ-5D | Not included | Not included |
| **Blake et al 2003 (18)** | Health care (though claimed to be social) | Screening and treatment: C-reactive protein test and 1 office visit /The mean of the average wholesale price for lovastatin (40 mg) and pravastatin (40 mg) and monthly dispensing fee; Patients receiving statins had the additional cost of two other office visits and two liver function tests in the first year of treatment. / CVD event: lifetime cost of MI (range given since this cost is age and gender specific), the acute cost of stroke, and the annual cost after stroke (range provided as this cost is age specific). | Previous published studies | 2000 US dollar | Age and sex-adjusted baseline data. Healthy : 0.90; post stroke: 0.74; post MI: 0.67 / no information about method used | Not included | Not included |
| **Pletcher MJ et al 2009 (21)** | Health care | Screening and treatment: Statin costs estimated by averaging the lowest average wholesale prices reported in national database, GP visit, lipid panel / management of side-effects. | Center for Medicare and Medicaid Services | 2006 US dollar | % reduction for being in the hospital = 50%, rhabdomyolisis of liver failure =20%, SA=8%, MI=15% cardiac arrest, 36% / no information about the method used | Not included | Yes |
| **Kok et al 2009 (22)** | Health care | Cost of diagnostic (only in the first year), doctor’s visits, repeat prescriptions, drug cost | Dutch cost of illness study | Year is not provided / euros | No information | Not included | Not included |
| **Shiffman et al 2012 (28)** | Health care | Cost of testing, cost of CVD event and cost of major GI bleeding event. | Several studies | 2009 US dollar | MEN: MI year 1=0.66 ; MI, subsequent years=0.76; Major GI bleeding:0.84;  WOMEN: MI year 1=0.58; MI, subsequent years=0.66; Major GI bleeding:0.67/ no information about the method used | Not included | Yes |
| **Choudhry et a 2011 (25)** | Social perspecive | Costs of high-sensitivity C-reactive protein test, liver function test, rosuvastatin*, treatment of cardiovascular events, and treatment of adverse events. The value of the time for both patients and informal care was considered using average hourly wages of age-matched US workers | Medicare payments, average wholesale prices, and published reports. Hospitalisation data were from the Nationwide Inpatient Sample and other resource use data were from published studies and databases | 2009 US dollar | Healthy:0.85  MI, UA:0.88  Stroke:0.73  Diabetes:0.88  End-stage renal disease:0.61 / no information about the method used | No reduction in base case. In sensitivity analysis include a utility decrement of 0.02 | Yes |
| **Lovibond et al 2011 (26)** | Health care | Costs of diagnosis (equipment, consumables, maintenance and staff time),  antihypertensive treatment^#^ (drugs and annual clinical review), and management of CVD. | Published reports and national estimates official NHS  sources | 2009-2010 UK | No event: 0.704-0.909 (age and sex dependent. Adverse events decrements applied multiplicatively to this general population baseline:  Stroke: 0.629; MI:0.760; UA:0.77; SA:0.808; TIA:1 /EQ-5D | Not included | Not included |
| **Marshall and Rouse 2002 (20)** | Primary health care | Nurse time, blood tests and drugs (including dispensing costs). | Personal Social Services Research Unit/ Pathology service/ British National formulary | Year is not provided , UK | Not included | Not included | Not included |
| **Lee et al 2010 (30)** | Health care (though claimed to be social) | Medication and laboratory testing/  MI (acute MI and post-MI) and Stroke (acute, post-stroke 1^st^ yr and subsequent) event /adverse event/Healthcare cost for healthy; End-of-life cost for each death | medicare reimbursement rates and literature reports | 2008, US dollar | Healthy: by age; Post MI:0.84  Post stroke:0.79 / EQ-5D | Utiility reduction between 1% and 2% | Mild adverse event reaction to statin: 2 days of lost life  Severe adverse event reaction to statin: 2 weeks of lost life |

**Notes: #:** Drug costs were based on the most commonly used generic drugs; *:The price of branded rosuvastatin was used, until the patent expired seven years into the simulation, when the generic price was used. GP: general practitioner; PBS: Pharmaceutical Benefits Scheme; CHF: CVD: cardiovascular disease; HRQL: health related quality of life; CHD: coronary heart disease; CIMT: carotid intima-media thickness; MI: myocardial infarction; EQ-5D: Euroqol 5 dimension; GI: Gastrointestinal; UA: unstable angina; SA: stable angina; TIA: transient ischemic attack

eTable 8. Validation and Sensitivity analyses

| **Study** | **Sensitivity Analysis / PSA** | **Effect of sensitivity analysis on the results** | **Validation and Method** |
| --- | --- | --- | --- |
| **Rapsomaniki et al 2011 (23)** | SA includes different treatment cost, efficacy and threshold. SA also includes a wide range of modelling assumptions: Different cross-validation approaches, alternative timescales, use of parametric hazards, use of study-specific hazards, different ways to evaluate benefit and different ways to combine study-specific estimates / no PSA | At a given treatment threshold, increasing treatment efficacy results in higher net benefits and higher difference in net benefit, despite the accompanying increase in treatment-related cost.  For a given treatment efficacy, cheaper interventions offered at low risk thresholds are much more cost-effective than expensive interventions targeted towards high risk individuals. In terms of cost-effectiveness comparisons, the gain from Model 2 appears to be scale-dependent, that is it increases with increasing net benefit from Model 1. | Validation is performed by using 10 fold cross validation. To explore the external validity of the models, they performed leave one-study-out cross-validation, whereby risk in each study is computed using parameters estimated from the remaining studies. Calibration is performed by assessing the correspondence between observed and predicted risk categories. |
| **Wald et al 2011 (24)** | One and two way SA: cost, adherence, discounting rate, effect of preventive treatment / no PSA | The most influential variable is the effect in treatment and the cost of preventive treatment. | Validation is performed by comparing the expected performance of age screening based on the expected age-specific incident of CVD event using FR equation with those observed from CVD registry data in England and Wales |
| **Cobiac et al 2012 (27)** | One way SA: blood pressure, smoking, diabetes and total cholesterol. / PSA | The overall cost-effectiveness of statins is highly dependent on the baseline risk factor values. The main source of uncertainty in this study is the ability of the 4S and FINRISK functions to predict the occurrence of CHD events in Finland. | No validation was described |
| **Johannesson 2001 (19)** | One way SA: of reduction in risk of CVD event, increase of mortality, and annual intervention cost (drugs, laboratory test and physician visit)/ no PSA | The result was most sensitive towards the variations in the risk reduction, the intervention cost, and the rate of discounting costs and QALYs. | No validation was described |
| **Ruitjer et al 2013 (29)** | Result by different scenarios: time horizon and optimistic scenario of RR due to treatment / PSA | Results are sensitive to the time horizon. When the cost of a CIMT and plaque measurement decreases, or increase the treatment effectiveness (optimistic scenario) the cost effectiveness of the CIMT strategy will become more favourable, in particular within a 10 year horizon in both men and women. At a WTP of $50,000, the probability that CIM is cost-effective is 93% for men within 30 years and, 87% for women within 10 years . | No validation was described |
| **Blake et al 2003 (18)** | A three-way SA of the baseline 10-year risk of coronary heart disease, the annual cost of statin therapy and the efficacy of statin therapy for the prevention of MI was performed for 58-year-old men and women. /No PSA | Screening became increasingly cost-effective for both men and women as the 10-year risk of CHD increased, the cost of statin therapy decreased, or the efficacy increased. C-reactive protein screening becomes cost saving if the annual cost of statin therapy is reduced to $500 with an efficacy of 45% or greater. | No validation was described |
| **Pletcher MJ et al 2009 (21)** | One way SA: risk and age threshold, cost and side effect of statin, potential disutility from taking a daily pill, adherence, and ineffectiveness of statin at the extreme of age. / PSA | Results were highly sensitive to the assumptions of the impact of LDL cholesterol level on the risk of CHD. | Model was calibrated to reproduce national data on risk factor distributions, and CHD events in the base year. Validation is performed by comparing the model’s results under a broad range of scenarios with data from studies, clinical trials, and surveys, obtained from public sources or by personal communication. |
| **Kok et al 2009 (22)** | One way SA: effects, discount rates, time horizon and compliance / No PSA | Results were sensitive to the time horizon and compliance. | No validation was described |
| **Shiffman et al 2012 (28)** | One way SA: cost of test and CVD event, QALY estimate, RR associated with the 2 SNP, adherence rate to aspirin use recommendations / PSA | Results were highly sensitive to the cost of the test | No validation was described |
| **Choudhry et a 2011 (25)** | One- and two-way SA: efficacy of treatment, the cardiovascular risk (FR score), and the drug costs / PSA | The base-case findings were robust to the variations considered in the sensitivity analyses. The probability of test-and-treat being cost-effective at a threshold of $50,000 per QALY was 94%. | No validation was described |
| **Lovinbond et al 2011 (26)** | One way SA: diagnostic cost, cost of hypertension treatment, cost of CV event, failure rates, time until diagnosis complete, sensitivity and specificity, treatment effect, CVD risk, Check-up frequency in those diagnosed as not hypertensive, utility/ PSA | PSA showed that continuous monitoring was the most cost-effective strategy in almost all simulations.  The one-way SA confirmed its superiority, except in two scenarios: when testing at home was assumed to be equally sensitive to continuous testing (home preferred), and when treatment for those who were falsely diagnosed with hypertension reduced their cardiovascular risk (clinic preferred). | No validation was described |
| **Marshall and Rouse 2002 (20)** | One way SA: prior prioritisation of the patients by estimated cardiovascular risk, prior knowledge of the patients' blood pressure, and the effects of not prescribing statins or ACE inhibitors /No PSA | Deterministic SA showed that more CVD could be prevented with the same health service resources by assessing only those patients pre-selected on the basis of a prior estimate of their risk of CVD. Primary care teams with an electronic record of their patients' BP could conceivably reduce the time for patient assessment to 5 minutes, thus favouring the JBR strategies. Statins and ACE inhibitors cost more than identifying and treating new patients, so strategies avoiding these may allow more disease to be prevented within available resources. | No validation was described |
| **Lee et al 2010 (30)** | One way SA: event probabilities, RR of CV events; utility decrement for the use of statin; adherence of statin therapy; cost of event and treatment / PSA | Model result were very sensitive to potential harms from statin therapy, including frequent low-intensity muscle soreness, diabetes mellitus, and liver failure. Increasing cost of statin would make hs-CRP more cost-effectiveness. Increasing threshold from 2 to 10mg/l make hs-CRP screening less favourable. PSA identified greatest uncertainty around ICER in the lowest risk group. | Validation is performed: model results agreed well with CVD mortality in USA and JUPITER study. Method used is not provided. |

SA: deterministic sensitivity analysis; PSA: probabilistic sensitivity analysis; M1 model 1; M2: model 2; FR: Framingham risk; CVD: cardiovascular disease; CHD: coronary heart disease; QALY: quality-adjusted life year; RR: relative risk; SNP: single nucleotide polymorphisms; ACE: An angiotensin-converting-enzyme; BP: blood pressure; JBR: Joint British recommendations: ICER: incremental cost-effectiveness ratio;
